# PINK1 deficiency exacerbates age-related bone loss in association with enhanced inflammatory adipocyte differentiation

**DOI:** 10.1016/j.gendis.2026.102163

**Published:** 2026-03-27

**Authors:** Min-Ji Sung, Hyun-Ju An, Hyunjeong Yeo, Sujin Choi, Min Heui Ha, Hye Yun Jeong, Jihyun Baek, Jung Sooyoung, So-Young Lee, Soonchul Lee

**Affiliations:** a Department of Orthopaedic Surgery, CHA Bundang Medical Center, CHA University School of Medicine, Seongnam 13496, South Korea; b Division of Nephrology, Department of Internal Medicine, CHA Bundang Medical Center, CHA University School of Medicine, Seongnam 13496, South Korea; c SL Bio, Inc., 120 Haeryong-ro, Pocheon-si, Gyeonggi-do 11160, South Korea

**Keywords:** Adipogenesis, Age-related bone loss, Inflammation, Osteogenesis, PINK1

## Abstract

Age-related bone loss contributes to frailty, fractures, and increased mortality in the elderly, yet current osteoporosis treatments have limited long-term efficacy. Mitochondrial dysfunction is a hallmark of skeletal aging, but its role in mesenchymal lineage commitment remains unclear. Here, we investigated PTEN-induced kinase 1 (PINK1), a serine/threonine kinase essential for mitophagy, in regulating bone mass and adipogenic differentiation during aging. Using *Pink1* knockout (*Pink1*^−/−^) mice, we found that *Pink1* deficiency exacerbated trabecular bone loss and promoted adipocyte hypertrophy in white adipose tissue. Calvarial pre-osteoblasts from *Pink1*^−/−^ mice displayed impaired osteogenic differentiation and increased adipogenesis, evidenced by reduced alkaline phosphatase (ALP) activity, mineralization, and osteogenic gene expression, alongside elevated lipid accumulation. *In vitro*, siRNA-mediated *Pink1* knockdown in 3T3-L1 cells triggered adipogenic, inflammatory, and oxidative stress responses, while suppressing mitochondrial function and respiration. Collectively, these findings reveal that PINK1 preserves skeletal integrity by maintaining mitochondrial homeostasis and restraining stress-induced adipogenic reprogramming. Our study identifies the PINK1–mitophagy axis as a potential therapeutic target for preventing age-related bone loss and preserving bone health.

## Introduction

Age-related bone loss, or osteoporosis, is a major clinical challenge due to its association with increased fracture risk, morbidity, and mortality.^1^, ^2^, ^3^ During aging, osteoblast precursor cells gradually lose osteogenic potential while increasingly committing to adipogenic differentiation, leading to reduced bone mass and skeletal fragility.^4^, ^5^, ^6^ Strategies that inhibit this adipogenic shift may help preserve bone quality in elderly populations.

In addition to increased marrow adiposity, visceral fat accumulation has been linked to osteoporosis. Cross-sectional studies report correlations between visceral adiposity, body mass index (BMI), and osteoporotic fracture risk in both men and women.^7^, ^8^, ^9^, ^10^, ^11^ These associations are thought to involve altered mechanical loading, insulin resistance, and chronic low-grade inflammation, but the underlying molecular mechanisms remain incompletely understood.^12^

Mitochondrial dysfunction is increasingly recognized as a key contributor to impaired mesenchymal stem cell (MSC) differentiation and metabolic imbalance in aging-related diseases.^13^^,^^14^ Mitophagy, a selective mitochondrial quality control mechanism, maintains mitochondrial integrity and regulates energy metabolism in various cell types, including adipocytes. Although mitophagy has been extensively studied in metabolic diseases and neurodegeneration, its role in directing osteogenic versus adipogenic fate in bone-forming cells remains largely unexplored.

PTEN-induced kinase 1 (PINK1) is a mitochondrial stress sensor that initiates mitophagy and is essential for maintaining mitochondrial function.^15^, ^16^, ^17^, ^18^ Here, we investigated the impact of *Pink1* deficiency on mitochondrial respiration and adipogenic differentiation in osteoblast precursor cells. Our results reveal that *Pink1*-silenced cells exhibit reduced oxidative phosphorylation, increased oxidative stress, and enhanced adipogenic differentiation, suggesting that mitochondrial impairment underlies the shift in MSC fate. These findings provide mechanistic insight into the metabolic basis of age-related adiposity in the bone microenvironment and highlight mitophagy as a potential regulator of skeletal aging and osteoporosis.

## Materials and methods

### Animals

Conventional Pink1 knockout mice were obtained from Dr. Xiaoxi Zhuang (Department of Neurobiology, University of Chicago). Male *Pink1*^*−/−*^ mice aged 2 months (young) and 18 months (aged) were used in this study, along with age- and sex-matched C57BL/6J wild-type littermate controls (*Pink1*^*+/+*^). Mice were allocated into four experimental groups: 2-month-old *Pink1*^*−/−*^, 18-month-old *Pink1*^*−/−*^, 2-month-old *Pink1*^*+/+*^, and 18-month-old *Pink1*^*+/+*^, with six mice per group (*n* = 6 or 8). All animals were housed under specific pathogen-free conditions in a controlled environment (22–24 °C, 55%–60% humidity, 12-h light/dark cycle) with ad libitum access to standard laboratory chow and water. All experimental procedures were approved by the CHA University Animal Care and Use Committee (IACUC No. 200012) and conducted in accordance with institutional guidelines.

### Micro-computed tomography (μCT) analysis

High-resolution μCT imaging was performed on distal femurs fixed in 10% neutral-buffered formalin using a SkyScan 1173 μCT system (Bruker, Aartselaar, Belgium). Scans were acquired at an effective pixel size of 6.04 μm, with an X-ray source set at 130 kV and 60 μA, and an exposure time of 500 ms. Image reconstruction was performed using NRecon software (Bruker) with identical reconstruction parameters and a fixed global threshold applied across all samples. Trabecular bone analysis was conducted in a defined region of interest (ROI) located in the distal femoral metaphysis, starting at a fixed distance proximal to the growth plate and extending proximally for a consistent number of slices. Quantitative morphometric parameters, including bone volume fraction (BV/TV, %), trabecular thickness (Tb.Th, mm), trabecular separation (Tb.Sp, mm), and trabecular number (Tb.N, 1/mm), were calculated using CT Analyzer software (Bruker). Three-dimensional reconstructions of trabecular architecture were generated for visualization.

### Histological analysis of epididymal white adipose tissue (eWAT)

Epididymal white adipose tissue (eWAT) was harvested from *Pink1*^*+/+*^ and *Pink1*^*−/−*^ mice, fixed overnight in 10% neutral-buffered formalin, and embedded in paraffin. Paraffin sections (10 μm thickness) were stained with Periodic Acid–Schiff (PAS) to facilitate clear visualization of adipocyte boundaries for morphometric analysis. The cross-sectional area of adipocytes was quantified using ImageJ software (National Institutes of Health, USA). On average, 521 adipocytes per animal (range: 211–800 cells) were analyzed to calculate the mean adipocyte cross-sectional area. Adipocytes were further categorized into size bins to assess distribution patterns within eWAT. Quantitative analysis was performed in a manner, and samples from at least five mice per group were included in the analysis.

### Cell culture

Mouse calvarial pre-osteoblasts^19^, ^20^, ^21^ were isolated from *Pink1*^*+/+*^ and *Pink1*^*−/−*^ mice at postnatal day 3 (P3)^22^, ^23^, ^24^ following established protocols,^25^ demonstrating osteogenic and adipogenic differentiation potential. Calvariae were minced, digested with 0.3% type I collagenase in αMEM without fetal bovine serum (FBS) at 37 °C for 2 h, filtered through a 100-μm strainer, and cultured in αMEM with 10% FBS and 1% penicillin/streptomycin (growth media, GM). Cells at passages 2–4 were used for experiments. Bone marrow-derived mesenchymal stem cells (BM-MSCs) and epididymal white adipose tissue–derived MSCs (eWAT-MSCs) were isolated from femurs/tibias and eWAT of *Pink1*^*+/+*^ and *Pink1*^*−/−*^ mice, respectively, following standard protocols with minor modifications. Adherent cells were expanded in αMEM with 10% FBS and 1% penicillin/streptomycin and used at passages 2–4. For comparative *in vitro* analyses, 3T3-L1 pre-adipocytes and MC3T3-E1 pre-osteoblasts were cultured under the same conditions in a humidified incubator at 37 °C with 5% CO_2_. All cell lines were maintained at early passages to retain undifferentiated characteristics.

### Adipogenic and osteogenic differentiation

For adipogenic differentiation, 3T3-L1 cells and eWAT-MSCs isolated from *Pink1*^*+/+*^ and *Pink1*^*−/−*^ mice were cultured in DMEM supplemented with 10% FBS. Cells were induced for 2 days with MDI medium containing 1 mM IBMX, 0.5 μM dexamethasone, and 10 μg/mL insulin, followed by maintenance in DMEM with 10% FBS and 2.5 μg/mL insulin from day 4. From day 6 to day 10, cells were cultured in DMEM with 10% FBS (adipogenic media, AM) only, with medium changes every 2 days. All cells were used at passages 2–4 to ensure early, undifferentiated characteristics. For osteogenic differentiation, calvarial pre-osteoblasts, BM-MSCs, and MC3T3-E1 cells were cultured in osteogenic medium containing 50 μg/mL l-ascorbic acid and 10 mM β-glycerophosphate (osteogenic media, OM) for 5 days, with the medium refreshed every 3 days. Cells were maintained at early passages (2–4) to preserve osteogenic potential, and all differentiation experiments were performed under standard culture conditions (37 °C, 5% CO_2_).

### ALP activity assay and Alizarin Red S (ARS) staining

After 5 days of osteogenic differentiation, calvarial pre-osteoblasts, BM-MSCs, MC3T3-E1 cells and eWAT-MSCs (passages 2–4) were washed with phosphate-buffered saline (PBS) and fixed with 95% ethanol for 10 min. Alkaline phosphatase (ALP) activity was assessed using NBT/BCIP Ready-to-Use Tablets (Roche Diagnostics, Mannheim, Germany) according to the manufacturer's instructions, and ALP-positive cells were visualized under a microscope (Olympus CKX41SF, Tokyo, Japan). Quantification of ALP activity was performed by measuring the absorbance at 405 nm after cell lysis. For mineral deposition, cells were maintained in osteogenic medium for 14 days, washed with PBS, and fixed with 95% ethanol for 1 h. Cells were then rinsed with PBS and stained with 40 mM Alizarin Red S solution (pH 4.2; Sigma–Aldrich, St. Louis, MO, USA) for 30 min. Stained mineral deposits were dissolved in 10% (w/v) cetylpyridinium chloride (Sigma–Aldrich), and the absorbance was measured at 540 nm to quantify mineralization. All assays were performed on early passage cells (passages 2–4) to ensure reproducibility and retention of osteogenic potential.

### Oil Red O staining

3T3-L1 cells, eWAT-MSCs, and pre-osteoblasts from *Pink1*^*+/+*^ and *Pink1*^*−/−*^ mice (passages 2–4) were induced to undergo adipogenic differentiation for 10 days. After differentiation, the cells were washed with PBS and fixed in 60% isopropyl alcohol. Cells were then stained with freshly filtered 0.3% Oil Red O solution in 60% isopropyl alcohol at 37 °C for 15 min. Five random fields per sample were imaged, and lipid droplet accumulation was quantified using a semi-quantitative method. The dye was extracted from stained lipid droplets with isopropanol, and absorbance was measured at 510 nm using a microplate reader (Sunrise Remote; Tecan Austria GmbH, Grödig, Austria). All experiments were performed on early passage cells to ensure reproducibility and retention of adipogenic potential.

### *Pink1* knockdown cell line construction

To achieve *Pink1* knockdown, 3T3-L1 cells were transfected with *si-Pink1* (Santa Cruz Biotechnology, Inc., Dallas, TX, USA) using G-fectin (Genolution Pharmaceuticals, Inc., Seoul, South Korea) and incubated for 48 h. After transfection, cells were maintained in proliferation medium (DMEM supplemented with 8% calf serum; Gibco, Thermo Fisher Scientific, Waltham, MA, USA; 1610159) until confluence. Once the cells reached 2 days post-confluency, the medium was replaced with MDI medium supplemented with 10% FBS to initiate adipogenic differentiation. On day 4 of differentiation, the medium was replaced with a medium containing 1 μg/mL insulin and 10% FBS, with cells cultured for an additional 10 days.

### Generation of *Pink1* knockout MC3T3-E1 and 3T3-L1 cell lines

MC3T3-E1 pre-osteoblasts and 3T3-L1 preadipocytes were transfected with a piggyBac-based CRISPR/Cas9 construct (pPB[CRISPR]-Puro-Cas9-U6>m*Pink1*) targeting the murine *Pink1* gene using PEI. A piggyBac transposase helper plasmid was co-transfected to facilitate genomic integration. After recovery, transfected cells were selected with puromycin (1–2 μg/mL) for 5–7 days until non-transfected cells were eliminated. Puromycin-resistant cells were expanded and validated as *Pink1* knockout lines by Western blot and qPCR, confirming the loss of PINK1 expression. These pooled knockout populations were used for subsequent functional assays.

### RNA preparation and quantitative reverse transcription-polymerase chain reaction (qRT-PCR)

Total RNA was extracted from 3T3-L1 cells, calvarial pre-osteoblasts, BM-MSCs, and eWAT-MSCs (passages 2–4) derived from *Pink1*^*+/+*^ and *Pink1*^*−/−*^ mice using TRIzol™ reagent (Invitrogen, Thermo Fisher Scientific, Waltham, MA, USA) according to the manufacturer's instructions. One microgram of RNA was reverse-transcribed using the SuperScript™ IV First-Strand Synthesis System (Invitrogen) to generate cDNA. qRT-PCR was performed on a CFX96 Touch Real-Time PCR Detection System (Bio-Rad Laboratories, Hercules, CA, USA) using AMPIGENE® qPCR Green Mix Lo-ROX (Enzo Life Sciences, Farmingdale, NY, USA). mRNA expression levels were normalized to Gapdh, and relative expression was calculated using the 2−ΔΔCt method. Primer sequences are listed in Supplementary Table 1. All experiments were performed on early passage cells to ensure reproducibility and retention of differentiation potential.

### Western blot analysis

Protein extracts were prepared from cultured 3T3-L1 cells, MC3T3-E1 cells, BM-MSCs, and eWAT-MSCs (passages 2–4) derived from *Pink1*^*+/+*^ and *Pink1*^*−/−*^ mice using standard RIPA lysis buffer supplemented with protease and phosphatase inhibitors (Roche, Mannheim, Germany). Equal amounts of protein were resolved by SDS–PAGE and transferred onto PVDF membranes. After blocking in 5% non-fat milk or BSA, the membranes were incubated overnight at 4 °C with primary antibodies. Membranes were then incubated with HRP-conjugated secondary antibodies for 1 h at room temperature. Protein signals were visualized using enhanced chemiluminescence (ECL, Bio-Rad), and β-actin served as a loading control. Detailed information for all primary and secondary antibodies, including host species, clone/type, supplier, catalog number, and dilution, is provided in Supplementary Table 2. All experiments were performed on early passage cells to ensure reproducibility and retention of differentiation potential.

### PINK1 rescue assay (recombinant PINK1 treatment)

To restore PINK1 signaling in *Pink1*-deficient cells, recombinant mouse PINK1 protein (R&D Systems, Minneapolis, MN, USA) was applied during adipogenic or osteogenic differentiation. MC3T3-E1 and 3T3-L1 cells (passages 2–4) were seeded and differentiated as described above. Recombinant PINK1 was added at a final concentration of 200 ng/mL at the initiation of differentiation and replenished every 48 h during medium changes. Control cells received vehicle treatment. At the indicated time points, mitochondrial function, mitophagy markers, and differentiation outcomes were analyzed using Western blot, immunofluorescence, and Seahorse metabolic assays. All experiments were performed on early passage cells to ensure reproducibility and retention of differentiation potential.

### RNA sequencing and analysis

3T3-L1 cells were treated with si-Pink1 or control siRNA, with three biological replicates per group. Total RNA was extracted using NucleoZOL (Macherey–Nagel, Germany) according to the manufacturer's instructions. RNA sequencing was commercially performed by EBIOGEN (Seoul, South Korea). Briefly, sequencing libraries were generated and sequenced on an Illumina HiSeq X10 platform, yielding approximately 4G paired-end reads per sample. Raw sequencing reads were processed using BBDuk for adapter trimming and quality filtering, and clean reads were obtained in FASTQ format. Reads were then aligned to the *Mus musculus* reference genome (mm10) and corresponding transcriptome using TopHat. Gene-level read counts were quantified for each sample, and differential gene expression analysis was performed using ExDEGA (EBIOGEN, Seoul, South Korea). Gene set enrichment analysis (GSEA) was subsequently conducted to identify significantly enriched biological pathways. All RNA-seq data have been deposited in the Gene Expression Omnibus (GEO) database under accession number GSE280972.

### Oxygen consumption rate (OCR) measurement

The OCR was measured using a Seahorse XFe96 Extracellular Flux Analyzer (Agilent Technologies, Santa Clara, CA, USA). The cells were seeded in an XFe96 cell culture microplate at a density of 10^3^ cells per well and allowed to adhere overnight. Before the assay, the growth medium was replaced with Seahorse XF Base Medium (Agilent Technologies) supplemented with 10 mM glucose, 1 mM pyruvate, and 2 mM l-glutamine. Cells were then incubated at 37 °C in a non-CO_2_ incubator for 1 h to equilibrate temperature and pH. The mitochondrial stress test was conducted by sequentially injecting the following compounds: 1 μM oligomycin (to inhibit ATP synthase), 1 μM FCCP (to uncouple oxidative phosphorylation and measure maximal respiration), and 0.5 μM rotenone/antimycin A (to inhibit the activities of complexes I and III and measure non-mitochondrial respiration). OCR readings were recorded at baseline and after each injection. Data were normalized to the cell number using the CCK-8 viability assay and analyzed using the Seahorse Wave software (Agilent Technologies).

### Statistical analyses

All data are presented as the mean ± standard error of the mean (SEM) from at least three independent *in vitro* experiments and *in vivo* studies, with biological replicates indicated in the Figure legends. Statistical analyses were performed using GraphPad Prism 10 (GraphPad Software Inc., San Diego, CA, USA). Comparisons between two groups (*e.g.*, *Pink1*^*+/+*^ versus *Pink1*^*−/−*^ mice or si-Pink1 versus control siRNA in 3T3-L1 cells) were performed using the non-parametric Mann–Whitney U-test due to the non-normal distribution of small sample sizes. For comparisons involving more than two groups (*e.g.*, *Pink1*^*+/+*^, *Pink1*^*−/−*^, *si-Pink1*, and control siRNA), the Kruskal–Wallis test followed by Tukey's post hoc analysis was used. Correlations between PINK1 expression levels and experimental outcomes were assessed using Spearman's rank correlation analysis. A *P*-value < 0.05 was considered statistically significant.

## Results

### *Pink1* deficiency exacerbates age-related bone loss

**Figure 1:**
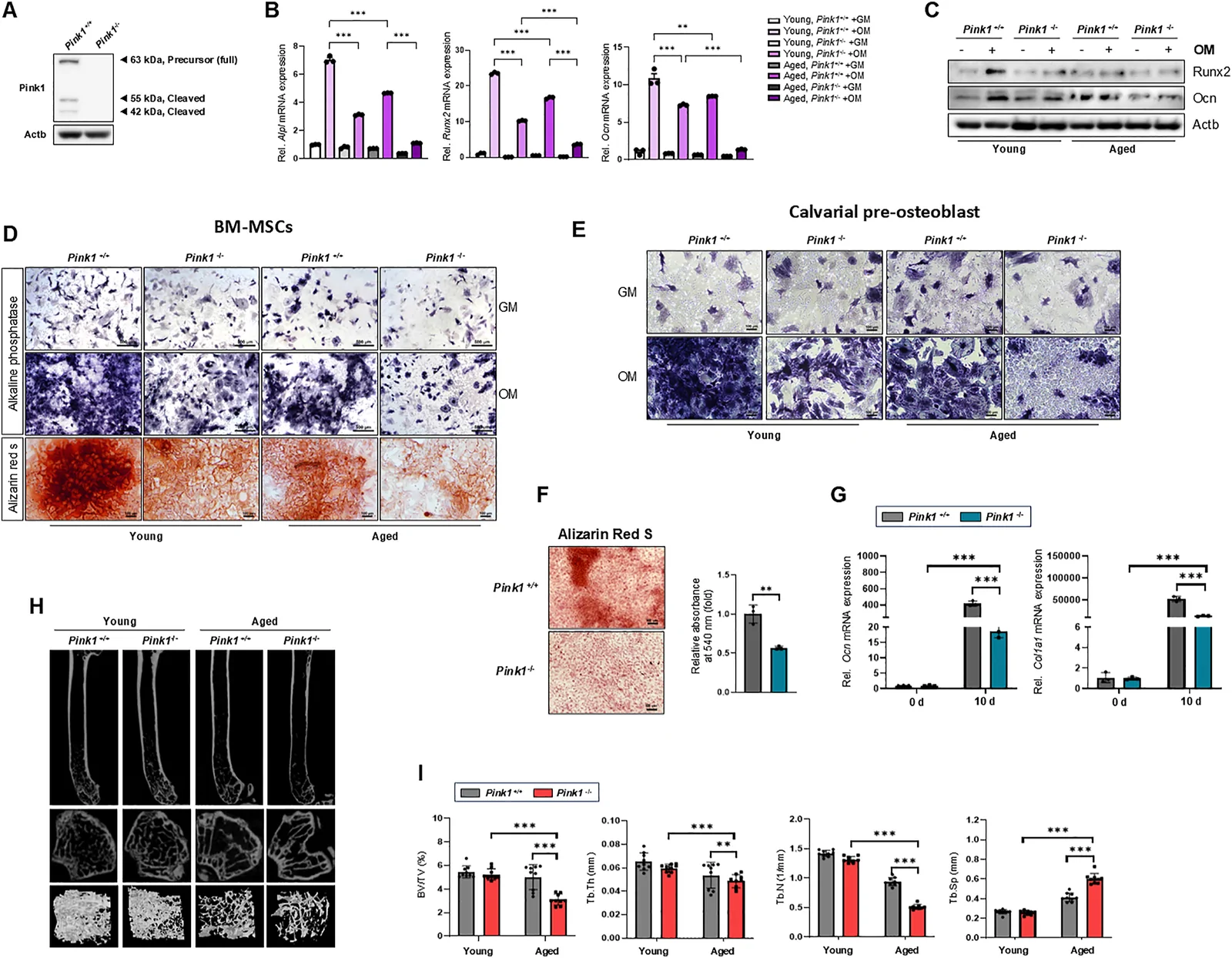
*Pink1* deficiency impairs osteogenic differentiation and accelerates age-related bone loss. **(A)** Western blot showing complete loss of PINK1 protein in femoral tissues from *Pink1*^−/−^ mice; β-actin served as a loading control. **(B)** qPCR analysis of osteoblast marker mRNA levels (*Alpl, Runx2, Ocn*) in femurs from young (2-month) and aged (18-month) mice. (*n* = 3 per group). **(C)** Western blot of osteogenic marker proteins in femoral tissues of experimental groups. (*n* = 5 per group). **(D)** Representative ALP and Alizarin Red S staining of osteogenically induced BM-MSCs, showing reduced differentiation in *Pink1*^−/−^ mice. Scale bars = 500 and 100 μm. **(E)** Calvarial pre-osteoblasts from *Pink1*^−/−^ mice displayed decreased ALP staining following osteogenic induction. Scale bars = 100 μm. **(F)** Alizarin Red S staining of calvarial pre-osteoblasts confirms impaired matrix mineralization under *Pink1* deficiency. Scale bars = 100 μm. **(G)** qPCR of late osteogenic markers (Ocn, Col1a1) in calvarial osteoblasts demonstrates significant suppression in *Pink1*^−/−^ cells. **(H)** Representative μCT images of femoral trabecular and cortical bone from young and aged *Pink1*^*+*/+^ and *Pink1*^−/−^ mice. Coronal, axial, and 3D-reconstructed views reveal compromised bone structure in *Pink1*-deficient mice. **(I)** Quantitative μCT morphometry of BV/TV, Tb.Th, Tb.N, and Tb.Sp. (*n* = 9 per group). Data are presented as mean ± SEM. ∗∗*p* < 0.01, ∗∗∗*p* < 0.001.

To investigate the role of PINK1 in skeletal aging, we analyzed *Pink1*^−/−^ mice and age-matched wild type (WT) controls. Western blot analysis confirmed complete loss of PINK1 protein in femoral bone tissue from *Pink1*^−/−^ mice (Fig. 1A). Under osteogenic induction, BM-MSCs isolated from both young (2-month) and aged (18-month) *Pink1*^−/−^mice exhibited markedly reduced expression of osteogenic markers—including *Alpl, Runx2, and Ocn*—at both the mRNA and protein levels (Fig. 1B and C). Consistently, ALP staining and Alizarin Red S assays revealed diminished early differentiation and mineralization capacity, with more pronounced defects in aged *Pink1*^−/−^ BM-MSC cultures (Fig. 1D). To determine whether these defects are conserved across mesenchymal progenitor sources, we next examined osteogenesis in calvarial pre-osteoblasts and eWAT-MSCs. Both calvarial and adipose-derived MSCs exhibited reduced ALP staining and mineral deposition in the absence of PINK1 (Fig. 1E and F; Fig. S1A), demonstrating lineage-independent suppression of osteoblast differentiation. Moreover, the expression of the late-stage osteogenic genes Ocn and Col1a1 was significantly downregulated in *Pink1*^−/−^ osteogenic cultures (Fig. 1G). These cellular defects translated into structural deterioration *in vivo*. Micro-CT analysis revealed that age-associated changes in trabecular architecture were modest in WT mice and did not reach statistical significance between young and aged WT animals within the examined time frame. Notably, no statistically significant differences in trabecular bone parameters were detected between WT and *Pink1*^−/−^ mice at the young age, indicating that structural bone alterations require additional time to manifest *in vivo*. In contrast, aged *Pink1*^−/−^ mice exhibited significant trabecular and cortical bone loss, characterized by reduced BV/TV, Tb.Th, and Tb.N, together with increased Tb.Sp (Fig. 1H and I). Together, these results establish PINK1 as an essential regulator of osteoblast differentiation and bone mass maintenance, and demonstrate that its deficiency accelerates age-related skeletal degeneration.

### *Pink1* deficiency enhances adipogenic lineage commitment and accelerates adipose expansion during aging

**Figure 2:**
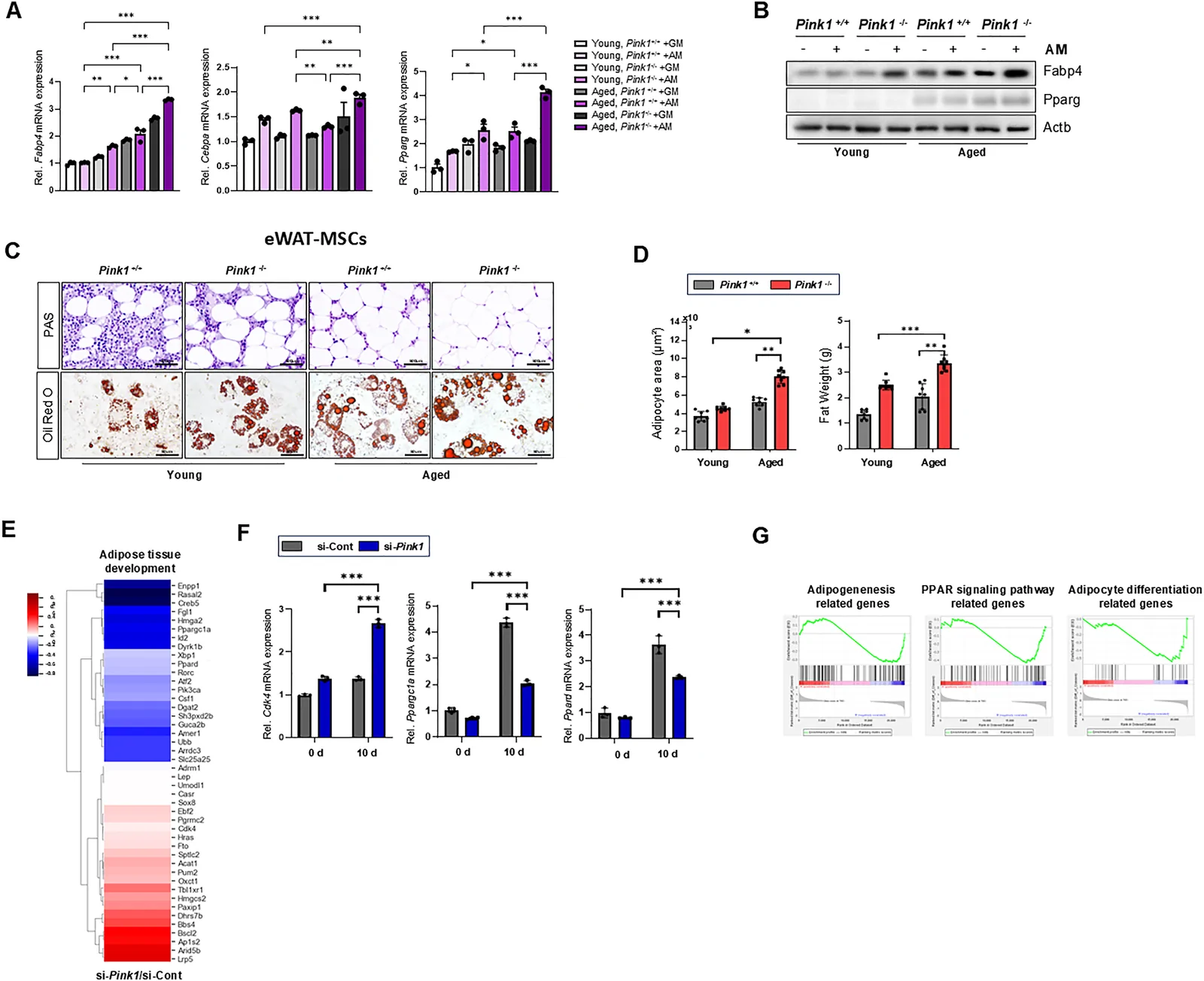
*Pink1* deficiency enhances adipogenic differentiation and promotes adipose tissue expansion. **(A)** qPCR analysis of adipogenic marker genes (*Fabp4, Cebpa*, and *Pparg*) in eWAT-derived MSCs (eWAT-MSCs) isolated from young (2-month) and aged (18-month) *Pink1*^+/+^ and *Pink1*^−/−^ mice following adipogenic induction. (*n* = 3 per group). **(B)** Western blot confirming increased adipogenic marker protein levels in *Pink1*-deficient cells. (*n* = 5 per group). **(C)** PAS staining of eWAT tissue sections shows enlarged adipocyte size in *Pink1*^−/−^ mice; Oil Red O staining of eWAT-MSCs demonstrates enhanced lipid droplet accumulation under adipogenic differentiation. Scale bars = 100 and 50 μm. **(D)** Quantification of adipocyte area and epididymal fat pad mass in *Pink1*^+/+^ and *Pink1*^−/−^ mice. (*n* = 8 per group). **(E)** Heatmap of adipogenesis- and lipid metabolism-related gene expression changes from RNA-seq in *siPink1*-treated 3T3-L1 preadipocytes. **(F)** qPCR validation in siPink1-treated cells showing upregulation of *Cdk4* and downregulation of Ppargc1a and Ppard during adipogenic differentiation. **(G)** Gene Set Enrichment Analysis (GSEA) plots illustrating activation of adipogenesis and PPAR signaling pathways in *Pink1*-deficient cells. Data are presented as mean ± SEM. ∗*p* < 0.05, ∗∗*p* < 0.01, ∗∗∗*p* < 0.001.

To determine whether *Pink1* deficiency biases adipogenic lineage commitment, mesenchymal stem cells isolated from epididymal white adipose tissue (eWAT-MSCs) of young (2-month-old) and aged (18-month-old) mice were subjected to adipogenic differentiation. qPCR analysis revealed a marked upregulation of the adipogenic transcription factors *Pparg* and *Cebpa*, indicating enhanced activation of the core transcriptional program governing adipocyte lineage commitment and differentiation. Moreover, the late adipogenic marker Fabp4, indicative of terminal adipocyte differentiation and lipid-handling capacity, was significantly increased in adipogenic-differentiated *Pink1*^−/−^ eWAT-MSCs, with a more pronounced elevation observed in aged mice (Fig. 2A). Western blot analysis further confirmed the increased protein abundance of these adipogenic markers (Fig. 2B), demonstrating that loss of PINK1 intrinsically promotes adipogenic differentiation of eWAT-MSCs. Consistently, PAS and Oil Red O staining demonstrated enhanced intracellular lipid droplet accumulation, a hallmark of mature adipocyte formation, in *Pink1*-deficient differentiated cultures (Fig. 2C). These findings suggest that enhanced adipogenic commitment of eWAT-MSCs may contribute to increased adipocyte formation capacity, thereby predisposing adipose tissue to expansion *in vivo*. *In vivo*, *Pink1*^−/−^ mice exhibited significantly enlarged adipocyte size and increased epididymal fat mass compared with age-matched controls (Fig. 2D), indicating accelerated adipose tissue expansion during aging. Mechanistically, RNA-seq profiling revealed strong enrichment of adipogenesis- and lipid metabolism-associated gene signatures upon *Pink1* knockout in 3T3-L1 preadipocytes (Fig. 2E). Supporting this, si*Pink1*-mediated knockdown increased *Cdk4*, a key regulator of mitotic clonal expansion during early adipogenesis, while suppressing *Ppargc1a* and mitochondrial oxidative gene programs, consistent with a metabolic shift favoring adipogenic differentiation (Fig. 2F). Notably, this transcriptional pattern is consistent with a shift toward early adipogenic commitment at the expense of mitochondrial oxidative capacity, rather than terminal differentiation per se. Gene Set Enrichment Analysis (GSEA) further confirmed the activation of PPAR signaling pathways under *Pink1*-deficient conditions (Fig. 2G). Collectively, these findings demonstrate that *Pink1* deficiency skews mesenchymal lineage commitment toward adipogenesis, thereby accelerating adipose tissue expansion during aging and contributing to a reciprocal imbalance between adipogenic and osteogenic differentiation, likely driven by impaired mitochondrial quality control.

### *Pink1* deficiency induces inflammatory and oxidative stress responses contributing to dysfunctional bone–fat microenvironments

**Figure 3:**
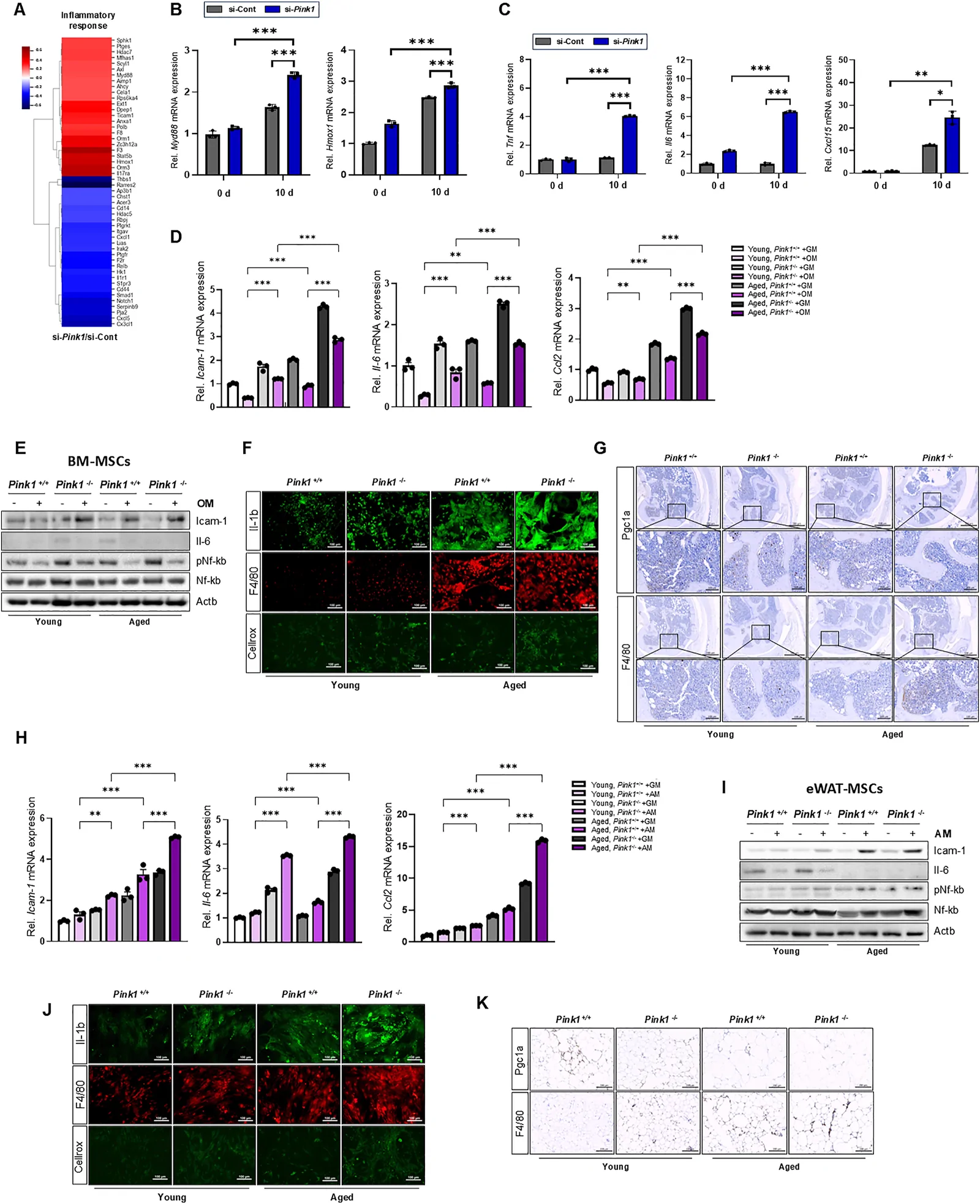
*Pink1* deficiency induces inflammatory activation and oxidative stress in bone–adipose tissue microenvironments. **(A)** RNA-seq heatmap of 3T3-L1 preadipocytes showing upregulation of inflammation-, chemotaxis-, and immune response-related gene signatures following *Pink1* depletion. (*n* = 3 per group). **(B)** qPCR validation of *Myd88* and *Hmox1* expression in *siPink1*-treated 3T3-L1 cells. **(C)** Expression of inflammatory cytokines (*Tnfα, Il-6, Cxcl15*) in *Pink1*^−/−^ eWAT-MSCs during adipogenic differentiation, with enhanced induction in aged mice. **(D)** qPCR analysis of *Icam1, Il-6*, and *Ccl2* in BM-MSCs during osteogenic differentiation under *Pink1*-deficient and aged conditions. **(E)** Western blot confirming increased activation of inflammation-associated pathways, including phosphorylated NF-κB (p–NF–κB), in *Pink1*^−/−^ cells. (*n* = 5 per group). **(F)** Immunofluorescence staining showing elevated IL-6, F4/80+ macrophage infiltration, and ROS accumulation detected by CellROX in differentiated cultures. Scale bars = 100 μm. **(G)** IHC of femoral bone sections showing decreased PGC-1α expression and increased F4/80+ macrophages in aged *Pink1*^−/−^ mice. Scale bars = 100 and 500 μm. **(H, I)** qPCR and Western blot analyses of eWAT-MSCs demonstrating progressive induction of inflammatory signaling during adipogenic differentiation under *Pink1* deficiency. (*n* = 5 per group). **(J)** Immunofluorescence staining for IL-6, F4/80, and CellROX confirming elevated inflammatory and oxidative stress levels in eWAT-MSC cultures. Scale bars = 100 μm. **(K)** IHC of epididymal adipose tissue showing reduced PGC-1α and increased F4/80+ macrophages in *Pink1*^−/−^ mice, indicating chronic adipose tissue inflammation *in vivo*. (*n* = 5 per group). Scale bars = 200 μm. Data are presented as mean ± SEM. ∗*p* < 0.05, ∗∗*p* < 0.01, ∗∗∗*p* < 0.001.

Adipocyte hypertrophy and lipid overload are known to trigger inflammatory cytokine production, macrophage recruitment and reactive oxygen species (ROS), thereby disturbing local tissue homeostasis.^26^, ^27^, ^28^ Given this reciprocal interaction between adipose inflammation and skeletal maintenance, we investigated whether *Pink1* deficiency elicits inflammatory and oxidative disruptions within bone–fat niches. RNA-seq analysis of 3T3-L1 preadipocytes revealed significant induction of inflammatory and chemotactic gene signatures upon *Pink1* depletion (Fig. 3A). Consistently, qPCR validation demonstrated increased expression of *Myd88* and *Hmox1*, indicating activation of innate inflammatory pathways mechanistically linked to suppressed osteogenesis and enhanced adipogenesis (Fig. 3B).^29^^,^^30^ These inflammatory stress responses provide a mechanistic bridge linking *Pink1* deficiency-driven metabolic dysfunction to lineage imbalance favoring adipogenesis. Pro-inflammatory cytokines, including *Tnfα, Il-6*, and *Cxcl15*, were also markedly upregulated in *Pink1*^−/−^ adipogenic cultures, with aging further amplifying these responses (Fig. 3C). A similar inflammatory phenotype was observed in BM-MSCs undergoing osteogenic differentiation, as Icam1, *Il-6*, and *Ccl2* expression was significantly elevated in the *Pink1*-deficient and aged groups (Fig. 3D), accompanied by enhanced phosphorylation of NF-κB (Fig. 3E). These findings suggest that PINK1 loss induces a shared pro-inflammatory milieu across both adipogenic and osteogenic compartments, thereby disrupting bone–fat microenvironmental homeostasis. Immunostaining of differentiated conditions confirmed stronger IL-6 signals, increased F4/80^+^ macrophages, and elevated ROS accumulation (Fig. 3F). *In vivo*, femoral bone sections from *Pink1*^−/−^ mice displayed reduced Pgc1a and increased F4/80^+^ macrophage infiltration, particularly in aged animals (Fig. 3G). Likewise, eWAT-MSCs exhibited sustained upregulation of inflammatory cytokines during adipogenic differentiation, supported by Western blot, qPCR and immunostaining analyses (Fig. 3H–J). Epididymal adipose tissue similarly showed decreased Pgc1a and increased macrophage infiltration under *Pink1* deficiency (Fig. 3K). Collectively, these data demonstrate that PINK1 loss drives chronic inflammation and oxidative stress in both bone and adipose microenvironments, thereby promoting adipose expansion while compromising skeletal integrity during aging.

### *Pink1* deficiency drives mitochondrial dysfunction and stress accumulation

**Figure 4:**
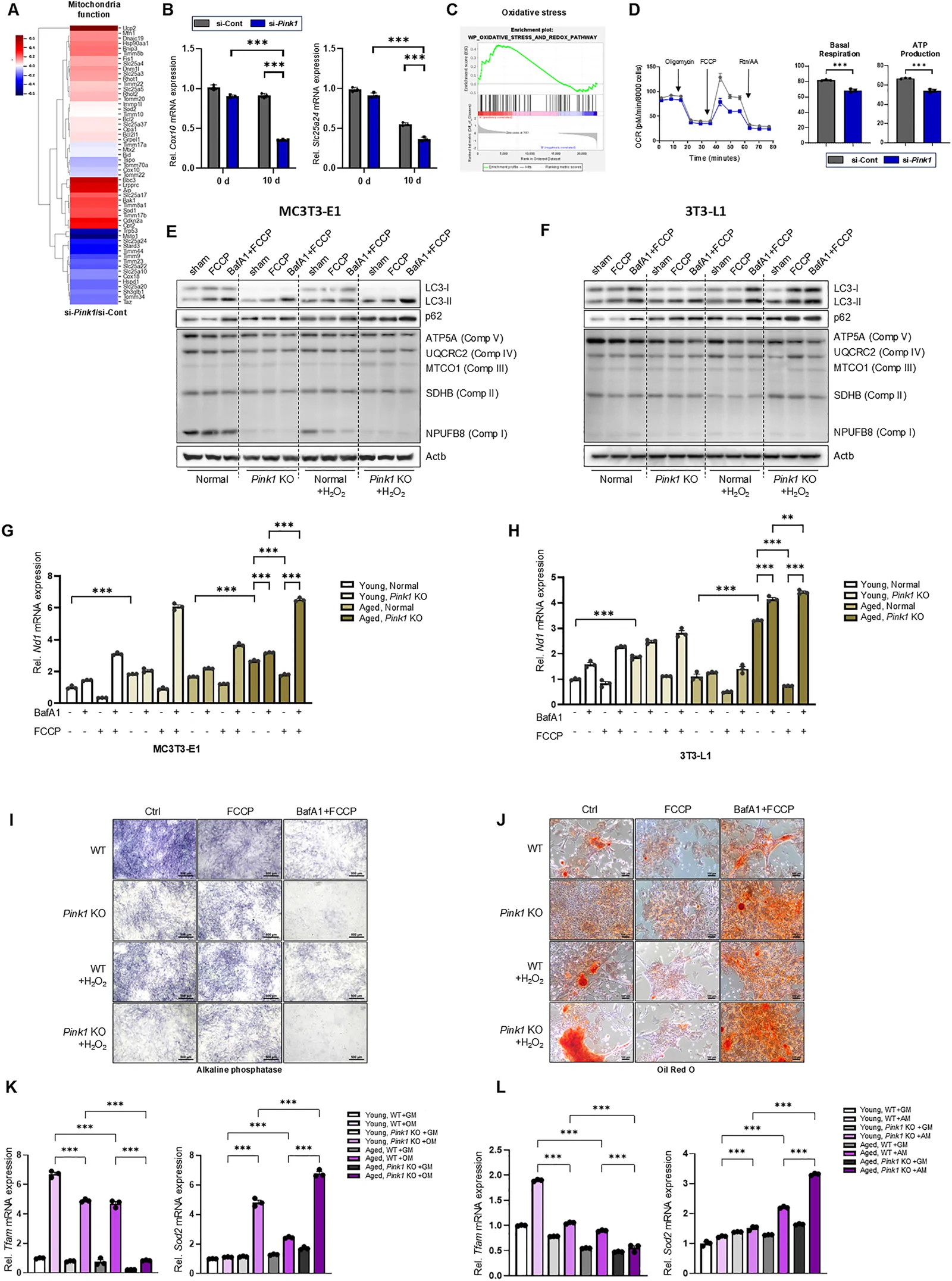
*Pink1* deficiency drives mitochondrial dysfunction and stress accumulation. **(A)** RNA-seq heatmap of 3T3-L1 cells showing global suppression of oxidative phosphorylation (OXPHOS) and mitochondrial metabolism-related genes upon *siPink1* knockdown. **(B)** qPCR analysis of mitochondrial function genes (*Cox10, Slc25a24*) at baseline (day 0) and after adipogenic differentiation (day 10), with further reduction under *siPink1* treatment. **(C)** GSEA plots showing enrichment of oxidative stress-associated pathways in *Pink1*-deficient cells following differentiation. **(D)** Seahorse XF analysis demonstrating reduced oxygen consumption rate (OCR), including basal and ATP-linked respiration, in *siPink1*-treated cells. **(E, F)** Western blot analyses in MC3T3-E1 and 3T3-L1 cells showing accumulation of LC3-II/I and p62 and decreased mitochondrial OXPHOS complex proteins, particularly Complex V, after FCCP or FCCP + BafA1 treatment in *Pink1*-deficient groups. (*n* = 3 per group). **(G, H)** mtDNA copy number analysis indicating the accumulation of dysfunctional mitochondria in *Pink1* knockdown cultures due to impaired mitophagy. (*n* = 3 per group). **(I)** ALP staining of MC3T3-E1 cells confirming impaired osteogenic differentiation under *Pink1* deficiency. Scale bars = 500 μm. **(J)** Oil Red O staining of 3T3-L1 cells showing enhanced lipid droplet accumulation after adipogenic differentiation in *Pink1*-deficient cells. **(K, L)** qPCR analysis of osteogenic (MC3T3-E1) and adipogenic (3T3-L1) cells showing decreased *Tfam* and increased *Sod2* expression, supporting mitochondrial dysfunction-driven redox imbalance. Scale bars = 100 μm. Data are presented as mean ± SEM. ∗∗*p* < 0.01, ∗∗∗*p* < 0.001.

Given that PINK1 is a key regulator of mitochondrial homeostasis, we next evaluated whether mitochondrial dysfunction underlies the impaired osteogenesis and increased adipogenesis observed under *Pink1*-deficient conditions. RNA-seq profiling of si*Pink1*-treated 3T3-L1 cells revealed global suppression of oxidative phosphorylation and mitochondrial metabolic pathways (Fig. 4A). qPCR further demonstrated that mitochondrial function-related genes (*Cox10, Slc25a24*) were reduced at baseline (day 0) and further decreased following adipogenic differentiation (day 10), particularly upon siPink1 treatment (Fig. 4B). Consistently, GSEA identified significant enrichment of oxidative stress-associated pathways after differentiation in *Pink1*-deficient cells (Fig. 4C). Functionally, Seahorse analysis showed markedly impaired mitochondrial respiration in si*Pink1*-treated cells, including significantly reduced basal and ATP-linked oxygen consumption (Fig. 4D). These bioenergetic defects provide a mechanistic basis by which *Pink1* deficiency constrains osteogenic energy demand while favoring adipogenic lineage commitment. In both MC3T3-E1 (osteogenic) and 3T3-L1 (adipogenic) models, mitochondrial uncoupling with FCCP—especially in combination with BafA1—exacerbated mitochondrial stress under *Pink1* deficiency, as demonstrated by elevated LC3-II/I and p62 alongside reduced OXPHOS complex protein abundance (Fig. 4E and F). Notably, FCCP increased LC3-II in both genotypes; however, p62 was reduced only in control cells, whereas *Pink1*-deficient cells exhibited p62 accumulation, indicating impaired autophagic flux and defective mitophagy. Importantly, OXPHOS decline was most pronounced in Complex V, reflecting the collapse of ATP-synthesizing capacity in *Pink1*-deficient cells. To assess mitochondrial turnover, mtDNA copy number was quantified and found to be significantly elevated in *Pink1*-deficient cultures (Fig. 4G and H). This increase in mitochondrial content likely reflects compensatory accumulation of dysfunctional mitochondria due to defective mitophagic clearance, rather than enhanced mitochondrial fitness. These mitochondrial abnormalities were functionally linked to reduced osteoblast performance, evidenced by decreased ALP activity and enhanced adipogenic lipid deposition (Fig. 4I and J). Because ROS elevation is known to drive adipogenic commitment by activating PPARγ and C/EBPβ signaling, we assessed redox regulatory genes. *Pink1* deficiency resulted in decreased *Tfam* and compensatory upregulation of *Sod2* (Fig. 4K and L), supporting mitochondrial dysfunction-driven redox imbalance. Collectively, these findings demonstrate that *Pink1* deficiency precipitates mitochondrial respiratory collapse and mitophagy impairment, leading to pathological accumulation of dysfunctional mitochondria that diminish osteogenic function while accelerating adipogenesis and oxidative stress-mediated degeneration during aging.

### *Pink1* deficiency drives mitophagy failure in mesenchymal progenitors

**Figure 5:**
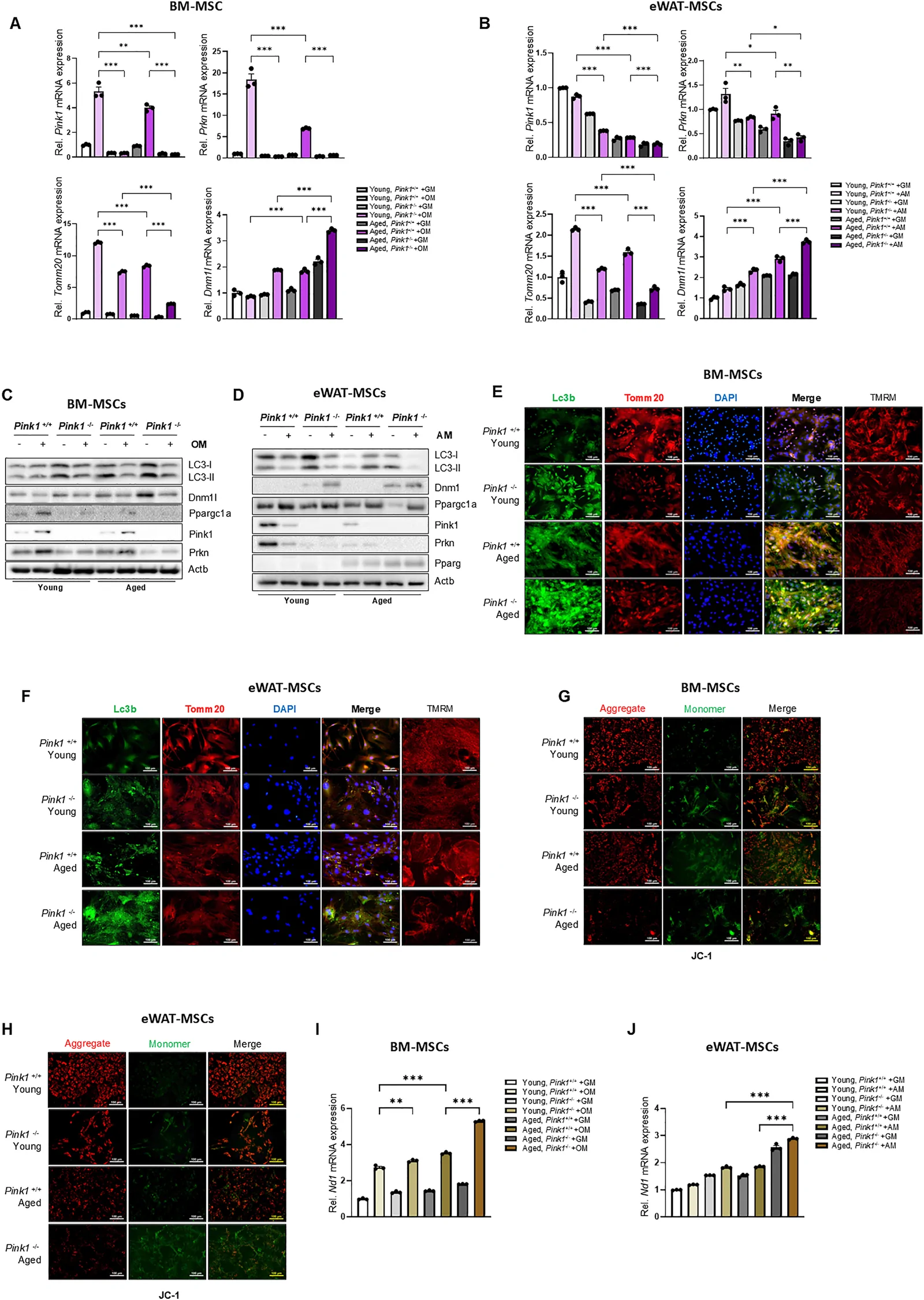
*Pink1* deficiency causes conserved mitophagy failure and mitochondrial dysfunction in primary mesenchymal progenitors. **(A, B)** qPCR analysis of mitophagy- and mitochondrial dynamics-related genes (*Pink1, Prkn, Tomm20, Dnm1l*) in BM-MSCs and eWAT-MSCs from young (2-month) and aged (18-month) *Pink1*^+/+^ and *Pink1*^−/−^ mice under growth medium conditions. (*n* = 3 per group). **(C, D)** Western blot showing decreased PINK1, PRKN, Dnm1l, and PGC-1α levels and increased LC3-II and DRP1 levels in *Pink1*-deficient MSCs. (*n* = 5 per group). **(E, F)** Immunofluorescence images of LC3B (green), TOMM20 (red), and TMRM (mitochondrial membrane potential indicator) showing increased mitochondria-associated LC3B puncta and reduced TMRM fluorescence in *Pink1*^−/−^ BM-MSCs and eWAT-MSCs. **(G, H)** JC-1 staining demonstrating enhanced mitochondrial depolarization (green monomers) and decreased polarized mitochondrial population (red aggregates) in *Pink1*-deficient MSCs. **(I, J)** Quantification of mtDNA copy number showing accumulation of damaged mitochondria in *Pink1*^−/−^ MSCs, particularly in aged mice. (*n* = 3 per group). Scale bars = 100 μm. Data are presented as mean ± SEM. ∗∗*p* < 0.01, ∗∗∗*p* < 0.001.

To determine whether mitochondrial dysfunction universally causes mitophagy defects under *Pink1* deficiency, we examined primary BM-MSCs and eWAT-MSCs isolated from *Pink1*^−/−^ mice. Pink1 loss markedly suppressed *Pink1*, *Prkn*, and *Tomm20* while upregulating *Dnm1l*, indicating impaired PINK1-PRKN-dependent mitophagy initiation and excessive mitochondrial fragmentation (Fig. 5A and B). Western blot analysis further confirmed reduced PINK1–PRKN signaling accompanied by accumulation of LC3-II, consistent with a blockade of mitophagic clearance rather than enhanced autophagy flux. Thus, increased LC3-II reflects stalled mitophagosome processing rather than productive mitochondrial turnover. Reduced PGC-1α expression further suggested compromised mitochondrial biogenesis and turnover in Pink1-deficient MSCs (Fig. 5C and D). Consistently, LC3B/TOMM20 co-immunofluorescence revealed pronounced accumulation of mitochondria-associated autophagosomes, particularly in aged *Pink1*^−/−^ cells (Fig. 5E and F). In addition, mitochondrial membrane potential assessment using TMRM staining demonstrated a marked reduction in fluorescence intensity in *Pink1*-deficient MSCs, indicating widespread mitochondrial depolarization. These findings were corroborated by JC-1 staining, which showed a prominent shift from red aggregates to green monomers in both BM-MSCs and eWAT-MSCs (Fig. 5G and H). Finally, mtDNA copy number was significantly elevated in *Pink1*-deficient MSCs, especially under aging conditions, indicating pathological accumulation of damaged mitochondria due to defective mitophagy (Fig. 5I and J). This failure of mitochondrial quality control provides a cellular basis for the impaired osteogenic capacity and enhanced adipogenic bias observed in *Pink1*-deficient mesenchymal progenitors during aging. Collectively, these findings demonstrate that *Pink1* loss disrupts PINK1–PRKN-dependent mitophagy across mesenchymal progenitors, leading to the accumulation of fragmented, depolarized, mtDNA-laden mitochondria that contribute to skeletal aging-associated degeneration.

### PINK1 rescue restores mitophagy and OXPHOS activity

**Figure 6:**
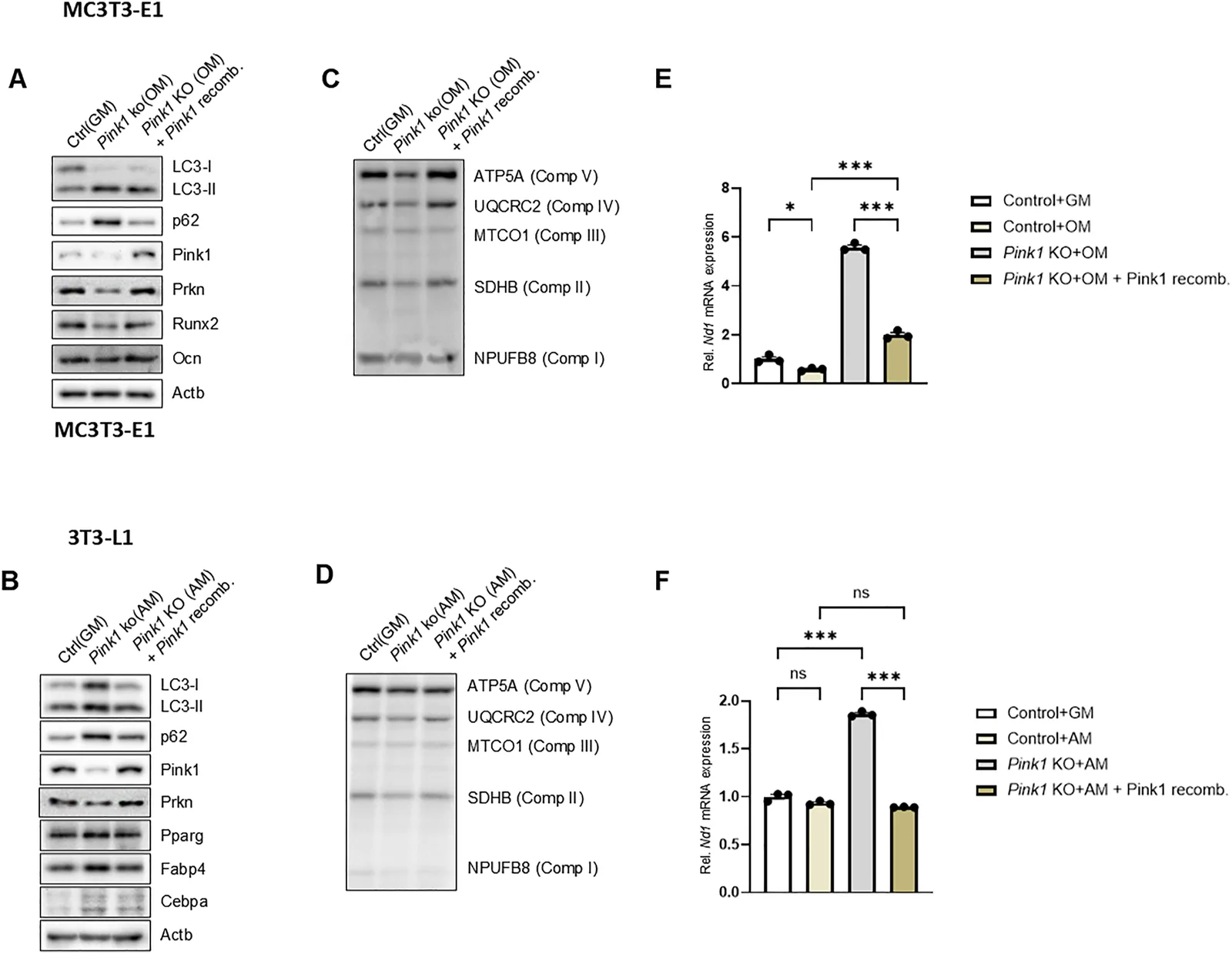
Recombinant PINK1 restores mitophagy flux and respiratory chain integrity in *Pink1*-deficient mesenchymal progenitors. **(A, B)** Recovery of mitophagy signaling in *Pink1* KO MC3T3-E1 and 3T3-L1 cells after recombinant PINK1 treatment, shown by reduced LC3-II and p62 levels and restoration of osteogenic or adipogenic marker expression. (*n* = 3 per group). **(C, D)** Western blot demonstrating recovery of mitochondrial OXPHOS complexes, particularly Complex IV and V, indicating improved electron transfer and ATP production upon PINK1 restoration. (*n* = 3 per group). **(E, F)** mtDNA copy number analysis showing a marked reduction in accumulated mitochondrial genomes in *Pink1* KO cells following recombinant PINK1 treatment, reflecting restored mitochondrial clearance. Data are presented as mean ± SEM. ∗*p* < 0.05, ∗∗*p* < 0.01, ∗∗∗*p* < 0.001.

To determine whether reactivation of PINK1 signaling restores mitochondrial quality control, we performed a rescue assay using recombinant PINK1 in *Pink1*-knockout MC3T3-E1 and 3T3-L1 cells. Restoration of PINK1 markedly reduced LC3-II accumulation and normalized p62 levels, demonstrating recovery of mitophagic flux (Fig. 6A and B). Importantly, recombinant PINK1 robustly rescued OXPHOS capacity, with a notable restoration of Complex IV (cytochrome c oxidase) and Complex V (ATP synthase) abundances—two critical respiratory complexes that were severely diminished in *Pink1*-knockout cells (Fig. 6C and D). Since Complex IV mediates the terminal step of electron transfer to oxygen and Complex V generates mitochondrial ATP, their recovery reflects a functional restoration of respiratory chain throughput and bioenergetic efficiency. These findings indicate that destabilization of Complex IV and V represents a key bioenergetic bottleneck underlying mitochondrial failure in the absence of PINK1. Consistently, mtDNA accumulation, which was previously elevated due to defective mitophagy, was substantially reduced following PINK1 replenishment (Fig. 6E and F), indicating enhanced clearance of damaged mitochondria and improved mitochondrial turnover. Restoration of mitochondrial quality control through PINK1 rescue therefore functionally links mitophagy recovery to normalization of mesenchymal cell fate and metabolic competence. Together, these findings confirm that *Pink1* deficiency induces mitochondrial respiratory collapse through loss of Complex IV and V stability, whereas PINK1 restoration reverses mitophagy and bioenergetic defects, thereby reinforcing PINK1 as a central regulator of mitochondrial homeostasis in mesenchymal progenitors.

## Discussion

Aging disrupts the homeostatic balance between osteogenesis and adipogenesis within the bone marrow niche, ultimately resulting in reduced skeletal strength and increased marrow adiposity. Because both bone-forming osteoblasts and lipid-storing adipocytes originate from a shared mesenchymal stem cell (MSC) pool, a shift in lineage allocation is considered a fundamental driver of skeletal aging.^31^, ^32^, ^33^ However, the upstream regulatory mechanisms that coordinate mitochondrial fitness with lineage specification remain poorly defined. In this study, we identified the mitochondrial serine/threonine kinase PINK1 as a central regulator that simultaneously preserves osteoblastogenesis, restrains excessive adipogenic conversion, and maintains mitochondrial and immune-metabolic homeostasis during aging.

Consistent with earlier reports that aging alone produces only modest bone loss in WT mice under physiological conditions,^34^^,^^35^ our results demonstrate that loss of PINK1 markedly accelerates the progression of skeletal deterioration over time. Although tissue-level structural changes were limited in young animals, genetic inactivation of Pink1 lowered the threshold for age-associated bone degeneration, resulting in pronounced trabecular and cortical bone deficits that became increasingly evident with aging (Fig. 1H and I). These findings indicate that the PINK1-mediated mitophagy pathway is not merely supportive but is essential for maintaining bone integrity by preventing the accumulation of cellular dysfunction that ultimately manifests as overt skeletal degeneration. Prior studies have demonstrated that PINK1/Parkin-dependent mitochondrial clearance promotes osteoblast survival and protects cells from metabolic and oxidative injury.^36^, ^37^, ^38^, ^39^, ^40^ In addition, PINK1-mediated mitophagy in osteoclasts has been shown to restrict inflammatory bone erosion.^41^^,^^42^ Importantly, our findings extend these observations by illustrating that systemic PINK1 loss induces intrinsic osteogenic defects at the cellular level before detectable tissue-level bone loss, and that these early defects progressively culminate in severe trabecular and cortical deterioration with aging (Fig. 1B–D, H, I). Together, these results position PINK1 as a crucial delaying factor in the onset and progression of skeletal frailty.

At the cellular level, we demonstrate that PINK1 is intrinsically required for osteogenic differentiation, as *Pink1*-null BM-MSCs, calvarial osteoprogenitors, and visceral adipose-derived MSCs uniformly exhibited impaired ALP activity, reduced mineralized matrix deposition, and downregulation of key osteogenic transcription factors. In stark contrast, *Pink1* deficiency markedly favored adipogenic lineage commitment, characterized by hypertrophic lipid accumulation in differentiated cultures accompanied by upregulation of PPARγ–C/EBPα signaling cascades. Transcriptome and GSEA analyses further revealed activation of adipogenesis-promoting metabolic programs in *Pink1*-deficient cells, establishing a direct mechanistic basis for the increased adipose burden observed in *Pink1*^−/−^ mice.

Moreover, a widely recognized pathological feature of skeletal aging is the reciprocal increase in marrow adiposity, which negatively impacts bone turnover and hematopoietic function. Indeed, clinical studies have shown that excessive bone marrow fat correlates with reduced trabecular bone volume and fracture risk.^7^^,^^43^^,^^44^ Although our *in vivo* adiposity assessments focused on epididymal fat and *ex vivo* differentiation models, the robust adipogenic drift we observed in *Pink1*-deficient MSCs strongly supports the likelihood of a similar deleterious shift in mesenchymal lineage composition within the aging skeleton. Thus, PINK1-dependent mitochondrial quality surveillance emerges as a key checkpoint that prevents maladaptive adipogenic takeover of the MSC pool. Mechanistically, our study provides compelling evidence that mitochondrial dysfunction is a central driver of these pathologic shifts. Loss of PINK1 provoked a severe decline in oxidative phosphorylation capacity, most notably through marked depletion of Complex IV and V—two respiratory chain components indispensable for electron transfer and ATP synthesis. Concomitantly, *Pink1* deficiency impaired mitophagic turnover of damaged mitochondria, leading to accumulation of TOMM20-positive depolarized organelles and elevated mtDNA content. Because osteogenic differentiation demands substantial ATP production and a metabolic transition toward oxidative phosphorylation, insufficient mitochondrial respiration directly limits osteoblast maturation, whereas glycolytic metabolic states are known to favor adipogenic identity. Our findings therefore support a bioenergetically driven lineage bifurcation, in which PINK1 loss biases MSCs toward lipid-rich phenotypes at the expense of bone-forming potential.

In addition to metabolic collapse, *Pink1* deficiency unleashed unrestrained inflammatory and oxidative stress responses across mesenchymal populations. This aligns closely with recent studies showing that PINK1 enhances the immunoregulatory functions of MSCs under oxidative challenge and suppresses inflammatory damage.^45^ We observed pronounced activation of *Myd88–Nf-κb* dependent cytokine programs, including *Tnf-α*, *Il-6*, and chemotactic mediators, which are well-established inhibitors of osteogenesis and potent promoters of adipogenesis.^29^^,^^30^^,^^46^^,^^47^

Heightened ROS accumulation and *Sod2* upregulation further indicated sustained redox stress, likely stemming from dysfunctional mitochondria.^36^^,^^41^^,^^48^, ^49^, ^50^ Thus, inflammaging and mitochondrial dysfunction appear to converge in the absence of PINK1, synergistically accelerating the pathologic replacement of bone with fat during aging.

This study primarily investigates PINK1 loss-of-function and does not evaluate PINK1 overactivation as a therapeutic countermeasure *in vivo*. In addition, because we used a global *Pink1* knockout model, we cannot fully exclude systemic or non-mesenchymal cell-autonomous contributions. PINK1 may differentially regulate mitochondrial quality control in osteoblasts, osteoclasts, adipocytes, and immune cells within the bone–fat microenvironment; therefore, bone- or adipocyte-specific conditional *Pink1* knockout and overexpression models will be essential to dissect cell-specific contributions. Additional studies using human MSCs or clinical bone samples from patients with mitochondrial dysfunction or metabolic disease will be important to validate translational relevance. Furthermore, defining upstream factors that reduce PINK1 expression during aging and resolving its crosstalk with osteoclasts and immune populations will deepen mechanistic insight. Future therapeutic investigations should include PINK1-stabilizing compounds, mitophagy-enhancing interventions, and exogenous recombinant PINK1 delivery to assess feasibility for clinical application.

Together, these findings establish PINK1 as a master regulator of mitochondrial quality control that safeguards skeletal integrity by suppressing inflammatory adipogenesis and maintaining osteoblast differentiation. Loss of PINK1 disrupts this metabolic and lineage balance, driving age-related bone degeneration and pathological fat expansion. Importantly, the ability to therapeutically rescue mitochondrial health and lineage balance through PINK1 reactivation provides functional evidence that PINK1 loss is causally linked to skeletal aging phenotypes.

## Conclusions

Our findings demonstrate that PINK1 is essential for maintaining skeletal integrity by preserving mitochondrial function and preventing adipogenic reprogramming in osteogenic precursors. These results identify the PINK1–mitophagy axis as a key regulatory mechanism underlying age-related bone loss and bone homeostasis.

## CRediT authorship contribution statement

**Min-Ji Sung:** Writing – review & editing, Writing – original draft, Formal analysis, Data curation. **Hyun-Ju An:** Writing – original draft, Formal analysis, Data curation. **Hyunjeong Yeo:** Writing – original draft, Formal analysis, Data curation, Conceptualization. **Sujin Choi:** Writing – review & editing, Formal analysis. **Min Heui Ha:** Writing – review & editing. **Hye Yun Jeong:** Methodology, Data curation. **Jihyun Baek:** Methodology, Formal analysis. **Jung Sooyoung:** Methodology. **So-Young Lee:** Writing – review & editing, Methodology, Investigation, Funding acquisition, Conceptualization. **Soonchul Lee:** Writing – review & editing, Methodology, Investigation, Funding acquisition, Conceptualization.

## Ethics declaration

The mice used in the present study were maintained according to guidelines approved by the Institutional Animal Care and Use Committee of CHA Medical University (IACUC; permit no. 220125).

## Funding

This work was supported by the National Research Foundation of Korea (NRF) grant funded by the Korea government (MSIT) (No. RS-2023-00210067, RS-2022-NR070174, RS-2022-NR070217).

## Conflict of interests

The authors declared no conflict of interests.
