# Supplementary material for: PINK1 deficiency exacerbates age-related bone loss in association with enhanced inflammatory adipocyte differentiation
Source: Genes Dis. 2026 Mar 27;14(1):102163. doi: 10.1016/j.gendis.2026.102163 (PMC13589082; doi:10.1016/j.gendis.2026.102163)
Supplement: Supplementary file 1 [file mmc1.docx]

Table S1. Mouse primer lists for qRT-PCR

| **Gene** |  | **Sequence (5’-3’)** | **Annealing temperature (°C)** |
| --- | --- | --- | --- |
| *Adipoq* | Forward | TGTTCCTCTTAATCCTGCCCA | 57,7 |
|  | Reverse | CCAACCTGCACAAGTTCCCTT | 61.3 |
| *Cebpa* | Forward | AGCCGAGATAAAGCCAAACA | 58.0 |
|  | Reverse | CCTTGACCAAGGAGCTCTCA | 56.5 |
| *Ccl2* | Forward | TTAAAAACCTGGATCGGAACCAA | 60.1 |
|  | Reverse | GCATTAGCTTCAGATTTACGGGT | 60.7 |
| *Col1a1* | Forward | TACACMTGGCCTAAGGGTCC | 60.9 |
|  | Reverse | TGTGGCAGATACAGATCAAGCA | 59.9 |
| *Cdk4* | Forward | CTGGTTTTGTCAGTATGTGTGCT | 57 |
|  | Reverse | TCCTCCATTAGGAACTCTCACAC | 58 |
| *Cox10* | Forward | AGAAGAGCTATACAGGGATTGCC | 63 |
|  | Reverse | CTGTGTGACATACATGCGCTT | 57 |
| *Cxcl15* | Forward | CGTCCCTGTGACACTAAGA | 58.4 |
|  | Reverse | TAATTGGGCCAACAGTAGCC | 56.9 |
| *Fasn* | Forward | GGAGCGCAGGATAGACTCAC | 59.0 |
|  | Reverse | ACCTTAGAGGCAGTGCAGGA | 59.5 |
| *Dnm1l* | Forward | GCGAACCTTAGAATCTGTGGACC | 60.3 |
|  | Reverse | CAGGCACAAATAAAGCAGGACGG | 61.5 |
| *Fabp4* | Forward | TGAAATCACCGCAGACGACAGG | 62.1 |
|  | Reverse | GCTTGTCACCATCTCGTTTTCTC | 59.2 |
| *Gapdh* | Forward | AGGTCGGTGTGAACGGATTTG | 61.6 |
|  | Reverse | GGGGTCGTTGATGGCAACA | 60.9 |
| *Hmox1* | Forward | AAGCCGAGAATGCTGAGTTCA | 57 |
|  | Reverse | GCCGTGTAGATATGGTACAAGGA | 63 |
| *Icam1* | Forward | GGCATTGTTCTCTAATCTCTCCG | 58.5 |
|  | Reverse | TGTCGAGCTTTGGGATGGTAG | 58.8 |
| *Il6* | Forward | AGTTGCCTTCTTGGGACTGA | 57.9 |
|  | Reverse | TCCACGATTTCCCAGAGAAC | 56.3 |
| *Myd88* | Forward | AGGACAAACGCCGGAACTTTT | 57 |
|  | Reverse | GCCGATAGTCTGTCTGTTCTAGT | 63 |
| *Ocn* | Forward | TGAGCTTAACCCTGCTTGTGA | 58.9 |
|  | Reverse | ATAGATGCGTTTGTAGGCCGT | 58.9 |
| *Nd1* | Forward | CTAGCAGAAACAAACCGGGC | 58.5 |
|  | Reverse | CCGGCTGCGTATTCTACGTT | 59.6 |
| *Ppard* | Forward | TCCATCGTCAACAAAGACGGG | 59 |
|  | Reverse | ACTTGGGCTCAATGATGTCAC | 57 |
| *Ppargc1a* | Forward | TATGGAGTGACATAGAGTGTGCT | 61 |
|  | Reverse | CCACTTCAATCCACCCAGAAAG | 61 |
| *Pparg* | Forward | TAAGTGCGATTGTACCCGGAC | 61.9 |
|  | Reverse | TTTGTAGCCATAGTCAGCATTGT | 60.0 |
| *Pink1* | Forward | TTCTTCCGCCAGTCGGTAG | 58.2 |
|  | Reverse | CTGCTTCTCCTCGATCAGCC | 59.3 |
| *Prkn* | Forward | ACAAATGCATCTGGAGGGGA | 58 |
|  | Reverse | TCAGGTCCACTCGTGTCAAG | 58.4 |
| *Runx2* | Forward | CCTGAACTCTGCACCAAGTCCT | 61.2 |
|  | Reverse | TCATCTGGCTCAGATAGGAGGG | 59.5 |
| *Slc25a24* | Forward | AGGCTTTCGGCAGATGGTAAA | 57 |
|  | Reverse | CCTTCCTCGGTAAGCAACTTCT | 61 |
| *Sod2* | Forward | TAACGCGCAGATCATGCAGCTG | 63.1 |
|  | Reverse | AGGCTGAAGAGCGACCTGAGTT | 62.6 |
| *Tnf* | Forward | CCACATCTCCCTCCAGAAAA | 55.8 |
|  | Reverse | AGGGTCTGGGCCATAGAACT | 58.9 |
| *Tfam* | Forward | GAGGCAAAGGATGATTCGGCTC | 60.7 |
|  | Reverse | CGAATCCTATCATCTTTAGCAAGC | 56.9 |
| *Tomm20* | Forward | TGACTACGAGAAGGGTGTGG | 57.8 |
|  | Reverse | GTCGGAAGCTTGGTCAGAAG | 57.6 |

CHA Bundang Medical Center, CHA University School of Medicine
