# Supplementary material for: PINK1 deficiency exacerbates age-related bone loss in association with enhanced inflammatory adipocyte differentiation
Source: Genes Dis. 2026 Mar 27;14(1):102163. doi: 10.1016/j.gendis.2026.102163 (PMC13589082; doi:10.1016/j.gendis.2026.102163)
Supplement: Supplementary file 2 [file mmc2.docx]

Table S2. Antibody lists for Western blot analysis

| **Target** | **Supplier** | **Dilution** | **Catalog No.** |
| --- | --- | --- | --- |
| Actb | Santa cruz | 1:3000 | sc-47778 |
| Dnm1l | Abcam | 1:1000 | ab184247 |
| Fabp4 | Abcam | 1:1000 | ab92501 |
| Icam-1 | Abcam | 1:1000 | ab109361 |
| Il-6 | Abcam | 1:1000 | ab290735 |
| Lc3b | Novus Biologicals | 1:1000 | NB100-2220 |
| Nf-kb | CST | 1:2000 | 8242 |
| Ocn | Abcam | 1:1000 | ab93876 |
| OXPHOS | Abcam | 1:2000 | ab110413 |
| p62 | Abcam | 1:2000 | ab109012 |
| Pink1 | Abcam | 1:1000 | ab216144 |
| pNf-kb | CST | 1:2000 | 3033 |
| Pparg | Abcam | 1:1000 | ab41928 |
| Prkn | Santa cruz | 1:1000 | sc-32282 |
| Runx2 | Santa cruz | 1:1000 | ab192256 |
