# Supplementary material for: PINK1 deficiency exacerbates age-related bone loss in association with enhanced inflammatory adipocyte differentiation
Source: Genes Dis. 2026 Mar 27;14(1):102163. doi: 10.1016/j.gendis.2026.102163 (PMC13589082; doi:10.1016/j.gendis.2026.102163)
Supplement: Supplementary file 3 [file mmc3.docx]

**Supplementary Figure 1. *Pink1* deficiency impairs osteogenic differentiation potential of calvarial osteoblasts and eWAT-MSCs.** (A) ALP staining of eWAT-derived mesenchymal stem cells (eWAT-MSCs) following osteogenic differentiation reveals progressive decreases in ALP activity with *Pink1* deficiency and aging. Scale bars = 500 μm. These results demonstrate reduced osteoblast differentiation capability in the absence of PINK1 in both young and aged skeletal environments.
